# Wastewater Metagenomics for Antimicrobial Resistance and Pathogen Surveillance: A Bibliometric Analysis

**DOI:** 10.3390/microorganisms14071583

**Published:** 2026-07-20

**Authors:** Yiran Zheng, Ningxuan Ma, Bingxuan Zhao, Yuhe Li, Yuxin Tian, Jinpu Liu, Yue Quan

**Affiliations:** 1 Department of Agricultural Resources and Environment, Yanbian University, Yanji 133002, China; zhengyiran0519@163.com (Y.Z.); 18744351618@163.com (Y.T.); 13844839352@163.com (J.L.); 2 Department of Geography and Ocean Sciences, Yanbian University, Hunchun 133300, China; 18304473626@163.com (N.M.); liyuhe1102@163.com (Y.L.); 3 Department of Biological, Geological, and Environmental Sciences, University of Bologna, Via Sant’Alberto 163, 48123 Ravenna, Italy; bingxuan.zhao@studio.unibo.it

**Keywords:** antimicrobial resistance, metagenomics, wastewater surveillance, one health, bibliometric analysis, CiteSpace

## Abstract

Wastewater systems are critical reservoirs where antibiotic resistance genes, antibiotic-resistant bacteria, and pathogens converge and disseminate into receiving waters, posing risks to ecosystems and public health. Metagenomics enables culture-independent surveillance of resistome and pathogens in wastewater. After the COVID-19 pandemic, the rapid expansion of wastewater-based epidemiological surveillance, together with growing emphasis on the One Health framework, has further promoted the integration of wastewater metagenomic monitoring with public-health surveillance strategies. However, no bibliometric study has systematically mapped the global research landscape at the intersection of metagenomics, wastewater systems, antimicrobial resistance, and pathogen surveillance. This study retrieved 1161 publications from the Web of Science Core Collection and used CiteSpace to conduct bibliometric analyses. From 2010 to 2025, annual publications increased from 1 to 219, with 72.7% of the total output concentrated between 2021 and 2025. China led in publication output but showed low betweenness centrality, whereas Australia and Sweden served as key intermediaries. Keyword analysis revealed a gradual thematic evolution from the basic detection of antibiotic resistance genes in activated sludge, through studies of dissemination mechanisms, to recent work on One Health and wastewater surveillance. Literature co-citation analysis showed that integration between environmental monitoring and public health literature remains limited, suggesting that the translation of metagenomic surveillance data into health risk assessment frameworks is still at an early stage. By mapping the field’s knowledge structure and gaps, this review highlights priorities for advancing wastewater-based Antimicrobial Resistance surveillance, including standardizing analytical methods, developing artificial intelligence-assisted resistome analysis, promoting equitable participation from underrepresented regions, and operationalizing One Health surveillance, thereby supporting the translation of wastewater monitoring into actionable public-health solutions.

## 1. Introduction

Antimicrobial resistance (AMR) is widely recognized as one of the most urgent global health threats of the twenty-first century. A landmark study estimated that bacterial AMR directly caused approximately 1.27 million deaths worldwide in 2019 and was associated with an additional 4.95 million deaths, with these numbers expected to rise further in the absence of coordinated interventions [1]. Although the clinical dimensions of AMR have received sustained attention, the mechanisms by which resistance determinants disseminate through environmental pathways remain comparatively underexplored [2]. Wastewater systems occupy a central position in this environmental dimension. Wastewater treatment plants (WWTPs) receive and concentrate antimicrobial residues, antibiotic-resistant bacteria (ARB), antibiotic resistance genes (ARGs), and mobile genetic elements (MGEs) from diverse sources, including hospitals, households, the pharmaceutical industry, and agriculture [2,3,4,5,6]. Although conventional treatment processes are effective in removing organic pollutants and traditional pathogens, they do not fully eliminate ARGs and MGEs, which can subsequently be discharged into receiving waters through treated effluents [7]. Within wastewater systems, the coexistence of sub-inhibitory concentrations of antimicrobials, diverse microbial communities, and MGEs creates favorable conditions for horizontal gene transfer (HGT), potentially amplifying resistance and enabling the transfer of ARGs from environmental microorganisms to clinically relevant pathogens [8]. Therefore, understanding the distribution, dissemination, and fate of AMR determinants and pathogenic microorganisms in wastewater systems is essential for developing evidence-based strategies to mitigate the wastewater-mediated spread of resistance [9,10,11].

Metagenomics, which involves the culture-independent sequencing and analysis of total genomic DNA extracted directly from environmental samples, has become a powerful tool for the comprehensive monitoring of microbial communities, ARGs, and pathogens in wastewater systems [12]. Unlike targeted approaches such as quantitative PCR (qPCR), which detect only predefined genes, shotgun metagenomic sequencing can simultaneously characterize the complete resistome, defined as the total repertoire of ARGs in the environment, microbial community composition, virulence factor profiles, and MGE-mediated dissemination pathways within a single analytical framework [13]. This untargeted, genome-wide analytical capacity has made metagenomics a preferred approach for large-scale wastewater AMR surveillance programs, including multinational collaborative initiatives that established the first global baseline of wastewater-associated antimicrobial resistance [14,15]. The continued decline in sequencing costs and the maturation of bioinformatic workflows for resistome annotation have further accelerated the application of metagenomic approaches in this field [16].

The COVID-19 pandemic further accelerated the development of wastewater surveillance [17]. Since 2020, many countries have rapidly established surveillance programs for detecting severe acute respiratory syndrome coronavirus 2 (SARS-CoV-2) in wastewater, demonstrating the feasibility of wastewater-based epidemiology (WBE) as a population-level public-health surveillance tool [18]. The expansion of WBE infrastructure has provided a practical foundation for AMR surveillance. At the same time, the growing emphasis on the One Health framework has positioned wastewater-based AMR surveillance at the intersection of environmental science, microbiology, public health, and veterinary medicine [19]. These developments suggest that metagenomic surveillance in wastewater systems may be shifting from primarily environmental characterization toward broader public-health applications.

Despite the rapid growth and increasing policy relevance of this research area, a comprehensive bibliometric analysis mapping its global landscape, knowledge structure, and thematic evolution is still lacking. Several bibliometric studies have examined related but distinct topics, including ARGs in wastewater treatment plant effluents and receiving waters [20], emerging contaminants in groundwater [21], microplastics in aquatic environments [22], and emerging contaminants in sludge [23]. However, none of these studies have specifically analyzed the intersection of metagenomics, wastewater systems, and AMR and pathogen surveillance. An early bibliometric study on wastewater metagenomics published by Garrido-Cardenas et al. in 2017 covered only literature published up to 2016 and did not focus on antimicrobial resistance or pathogen surveillance [24]. Given the rapid expansion of the field in recent years, a substantial update and a more focused analysis are warranted.

This study aims to fill this gap by conducting a systematic bibliometric analysis of the global research landscape on metagenomic surveillance of AMR and pathogenic microorganisms in wastewater systems from 2010 to 2026. Based on 1161 publications retrieved from the Web of Science Core Collection and analyzed using CiteSpace, this study addresses the following research questions. First, what is the overall growth trajectory of this field, and can distinct developmental stages be identified? Second, which countries, institutions, and authors constitute the main research forces, and what are the key features of collaboration patterns in this field? Third, what is the disciplinary composition of this field, and to what extent does it demonstrate interdisciplinary integration? Fourth, what are the major research themes, and how have they evolved over time? Fifth, what are the current research frontiers and emerging directions? Sixth, what is the knowledge base of this field, and which foundational studies have shaped its development? Across these questions, this study also examines an integrative issue, namely whether the field is shifting from primarily environmental characterization toward public-health surveillance applications.

## 2. Materials and Methods

### 2.1. Data Sources and Search Strategy

The bibliographic data used in this study were obtained from the Web of Science Core Collection (WoS CC) through the Science Citation Index Expanded (SCI-Expanded) database. The Web of Science Core Collection (WoS CC) was selected as the primary database because it provides standardized bibliographic records, cited-reference information, and broad indexing coverage of international journals in environmental science, microbiology, and public health. In addition, its structured export format is compatible with CiteSpace-based bibliometric analysis [21,25,26]. The literature search was conducted on 22 May 2026, and the initial search yielded 1331 records. The following Boolean search string was applied to the topic search (TS) field.

TS = ((“metagenomic*” OR “metagenome*” OR “shotgun sequencing” OR “shotgun metagenomic*”) AND (“wastewater” OR “sewage” OR “wastewater treatment” OR “WWTP” OR “sewage treatment” OR “wastewater effluent” OR “wastewater influent” OR “activated sludge” OR “sewage sludge”) AND (“antibiotic resistance” OR “antimicrobial resistance” OR “antibiotic resistance gene*” OR “ARG” OR “ARGs” OR “resistome” OR “antibiotic-resistant bacteria” OR “ARB” OR “multidrug resistan*” OR “AMR” OR “pathogen*” OR “pathogenic bacteria” OR “pathogenic microorganism*” OR “virulence” OR “virulence factor*”))

The search strategy was designed to maximize retrieval sensitivity rather than specificity alone because terminology related to wastewater metagenomics, antimicrobial resistance, and pathogen surveillance has evolved rapidly and varies among disciplines. Therefore, retrieved records were subsequently screened according to predefined inclusion and exclusion criteria.

### 2.2. Inclusion and Exclusion Criteria

**Figure 1:**
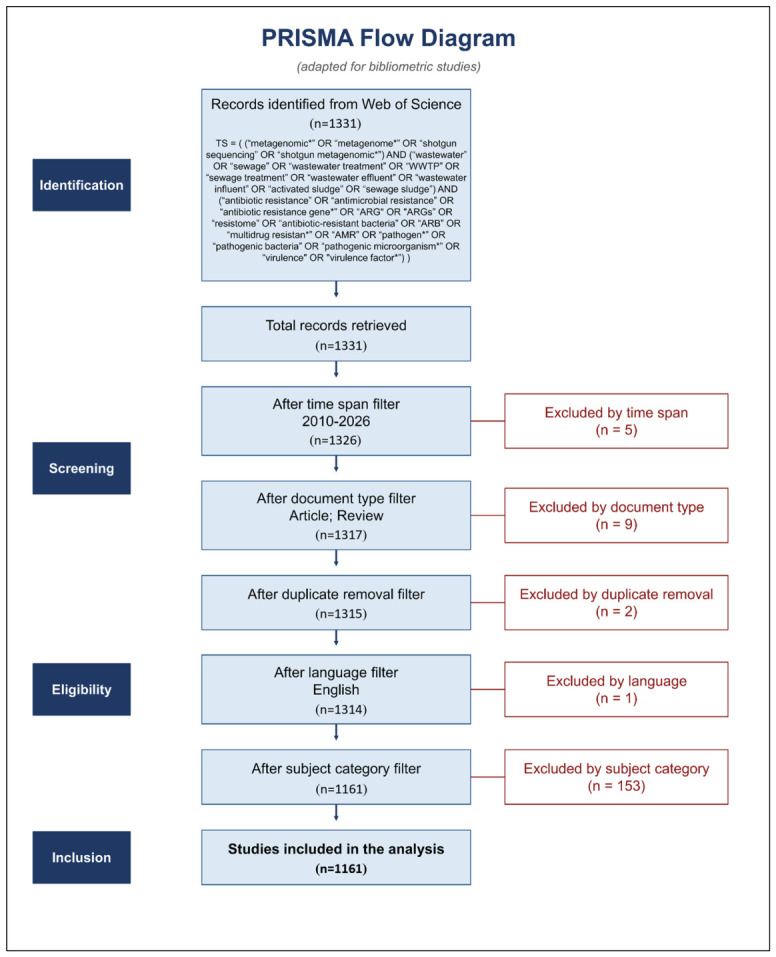
Exclusion flow diagram.

The retrieved records were screened sequentially, as shown in Figure 1. The publication period was restricted to 2010 to 2026, resulting in the exclusion of 5 records and leaving 1326 records. Document types were limited to articles and reviews, excluding 9 records and leaving 1317 records. After duplicate removal, 2 records were excluded, leaving 1315 records. Non-English publications were then removed, excluding 1 record and leaving 1314 records. Subject-category filtering and relevance screening were further applied to exclude records outside the scope of this study. Records were excluded if they did not investigate wastewater-associated ARG or pathogen surveillance, did not apply metagenomic approaches, or were outside the predefined research scope. This screening step excluded 153 records, yielding a final dataset of 1161 publications, including 1075 articles and 86 reviews, which accounted for 92.6% and 7.4% of the dataset, respectively.

Two reviewers (Yiran Zheng and Bingxuan Zhao) independently screened the titles and abstracts of the retrieved records. Full texts were consulted when eligibility could not be determined from the bibliographic information alone. Original research articles were eligible if they examined wastewater, sewage, activated sludge, sludge, influent, or effluent and applied shotgun metagenomics or another untargeted metagenomic approach to characterize antimicrobial resistance determinants, resistomes, pathogens, virulence factors, or related microbial features. Review articles were eligible when wastewater metagenomics for antimicrobial resistance or pathogen surveillance constituted a substantive focus. Records were excluded if they focused on non-wastewater matrices, used only targeted PCR/qPCR, 16S rRNA amplicon sequencing, or culture-based methods without a metagenomic component, or mentioned metagenomics, antimicrobial resistance, or pathogens only incidentally. Disagreements were resolved through discussion and, when necessary, adjudication by a third reviewer (Yue Quan). No record was excluded solely on the basis of its Web of Science subject category.

In addition, the data for 2026 were incomplete because the literature search was conducted on 22 May 2026. The 134 records published in 2026 were included in network construction but excluded from growth-rate calculations and stage-based analyses.

### 2.3. Data Export and Preprocessing

All 1161 records were exported from WoS in plain-text format, with the export option set to “Full Record and Cited References”. Exported data records can be found in the Supplementary Material. Author keywords were extracted from the Web of Science “Author Keywords (DE)” field and retained in their original indexed forms during network construction to preserve the information provided by the original publications. However, author-assigned keywords may contain lexical variations, including singular/plural forms, abbreviations, and alternative spellings. Therefore, semantically related terms were interpreted cautiously during thematic analysis rather than being considered as independent research directions solely based on differences in wording. For example, closely related terms associated with antibiotic resistance genes and wastewater terminology were synthesized at the broader conceptual level when discussing research themes. This approach avoids introducing subjective modifications to the original co-occurrence network after data extraction, while acknowledging that incomplete keyword harmonization may influence the fragmentation of individual keyword nodes.

### 2.4. CiteSpace Parameter Settings

CiteSpace (v.6.2.R4) was selected as the primary analytical tool based on the following methodological considerations. First, CiteSpace integrates co-citation analysis, burst detection, and timeline visualization within a single platform [25]. Second, its Pathfinder network pruning algorithm generates a more parsimonious network topology than the default algorithms used in tools such as VOSviewer. Third, the log-likelihood ratio (LLR) clustering algorithm provides statistically supported thematic labels. Although CiteSpace does not provide the overlay visualization available in VOSviewer or the thematic evolution mapping available in Bibliometrix, its integrated analytical workflow is best suited to the multidimensional analyses required in this study. Microsoft Excel was used to summarize annual publication trends.

The parameter settings were as follows. The time slicing was set from 2010 to 2026, with one year per slice. The g-index was selected as the node selection criterion because it emphasizes highly cited and influential items while allowing the inclusion of emerging but sufficiently impactful nodes. The setting of parameter k considers the balance between network readability and information retention, so different values are used when building different networks. The k values selected by different networks can be found in the upper left corner of the picture. Network pruning was performed using the Pathfinder algorithm. Cluster labels were generated using the LLR method. CiteSpace 6.2.R4, 64-bit Advanced Edition, was used for all analyses.

Modularity Q and weighted mean silhouette S were used specifically to evaluate the quality of keyword clustering generated by the LLR algorithm. For descriptive collaboration and co-citation networks, network structure was characterized using node number, link number, density, connected components, frequency, and betweenness centrality.

Keyword burst detection was performed in CiteSpace using Kleinberg’s burst-detection algorithm. The analysis used author keywords as the node type, a time span of 2010–2026, and one year per time slice. The burst-detection parameter γ was set to 1, and the minimum burst duration was set to 2 year(s). The 25 keywords with the strongest burst intensities were retained for visualization. Because the search was conducted on 22 May 2026, bursts extending to 2026 were interpreted cautiously as being based on partial-year data.

### 2.5. Bibliometric Analysis Workflow

The analysis comprised three complementary dimensions, including collaboration network analysis, thematic analysis, and knowledge-base analysis. Collaboration network analysis included co-occurrence networks of countries, institutions, authors, and disciplines. Thematic analysis included keyword co-occurrence, clustering, timeline analysis, and burst detection. Knowledge-base analysis included co-citation analyses at the levels of authors, journals, and individual documents. In addition, node frequency in co-citation networks represents co-citation counts rather than direct publication output. These two metrics have different meanings and were distinguished in the interpretation of the results.

## 3. Results

### 3.1. Publication Trend Analysis

**Figure 2:**
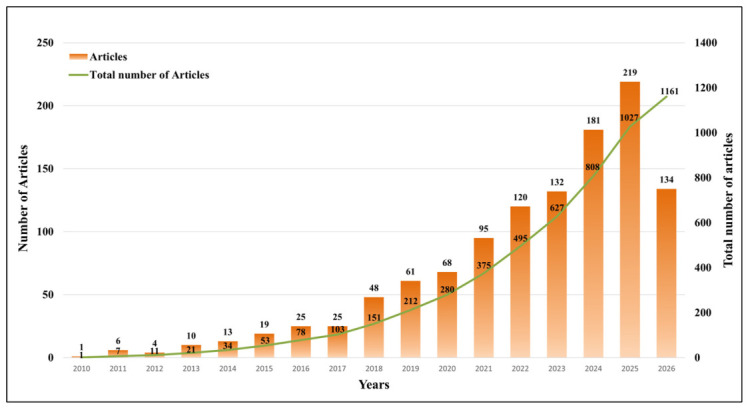
Annual and cumulative publication trends in research on metagenomic surveillance of antimicrobial resistance and pathogenic microorganisms in wastewater systems (2010–2026). Orange bars represent the number of articles published each year (left y-axis), and the green line represents the cumulative number of articles (right y-axis). Numerical labels above each bar indicate the annual count; numerical labels on the cumulative curve indicate the running total. Data for 2026 are incomplete, as the literature search was conducted on 22 May 2026, and therefore represent a partial-year count.

A total of 1161 publications were retrieved for the period from 2010 to May 2026. The annual number of publications increased from 1 in 2010 to 219 in 2025 (Figure 2).

For descriptive purposes, the publication trajectory from 2010 to 2025 was organized into three temporal periods based on the observed pattern of annual publication output. These periods were used to summarize broad changes in publication activity and were not derived from formal breakpoint analysis. The first stage, from 2010 to 2017, was an initial exploratory phase, during which 103 papers were published, with an annual average of approximately 12.9 papers, accounting for 10.0% of the total output for complete publication years (*n* = 1027). The second stage, from 2018 to 2020, was a period of steady development, marked by a sharp increase from 25 publications in 2017 to 48 in 2018; over these three years, 177 papers were published, with an annual average of 59.0 papers, approximately 4.6 times that of the first stage and accounting for 17.2% of the total output. The third stage, from 2021 to 2025, was a period of rapid growth, with annual publications increasing from 68 in 2020 to 95 in 2021, representing a 39.7% increase, and then continuing to rise to 219 in 2025; this stage produced 747 papers over five years, with an annual average of 149.4 papers, accounting for 72.7% of the total output. The 2026 data, comprising 134 publications as of 22 May, were not included in the stage classification, but already exceeded the full-year output of every year before 2022.

### 3.2. Country and Regional Collaboration Network Analysis

**Figure 3:**
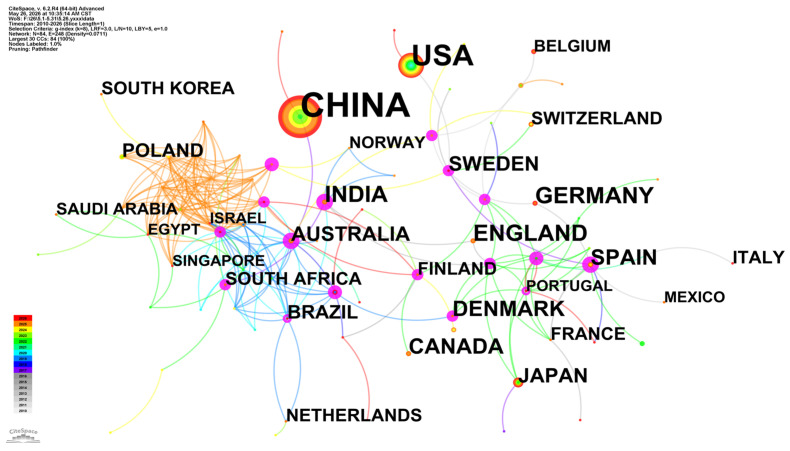
Country/region collaboration network in research on metagenomic surveillance of antimicrobial resistance and pathogenic microorganisms in wastewater systems (2010–2026). Node size is proportional to publication frequency; node colors reflect the year of first appearance, ranging from cool tones (earlier years) to warm tones (recent years). Purple rings indicate betweenness centrality greater than 0.1. Links represent co-authorship relationships between countries. The network comprises 84 nodes and 248 links (density = 0.0711, largest connected component = 84 nodes, 100%).

The country and regional collaboration network comprised 84 nodes and 248 links, with a network density of 0.0711 (Figure 3). All nodes formed a single connected component, indicating that all active countries were connected through direct or indirect collaborative relationships.

China was the most productive country, contributing 571 publications, followed by the United States (218), the United Kingdom (66), India (65), and Germany (61). The top 15 countries were distributed across East Asia, North America, Western and Northern Europe, South Asia, and South America, reflecting the broad geographical coverage of this research field. However, the distribution was markedly uneven, with China alone accounting for approximately 49% of the total output and China and the United States together accounting for approximately 68%.

Betweenness centrality analysis revealed a marked divergence between publication output and network-bridging roles. Australia (centrality = 0.37), Sweden (0.30), Spain (0.22), India (0.21), Brazil (0.19), and Finland (0.18) showed the highest betweenness centrality values, indicating that they occupied structural intermediary positions in connecting research communities across countries. In contrast, despite their dominant publication output, China and the United States both had betweenness centrality values of only 0.05, suggesting that their collaborations were concentrated within a relatively limited set of partner countries rather than being broadly distributed across the network. Publications indexed in the analyzed Web of Science corpus showed limited representation from Africa and the Middle East, with only South Africa ranking among the top 20 countries, with 23 publications and a centrality value of 0.11.

### 3.3. Institutional Collaboration Network Analysis

**Figure 4:**
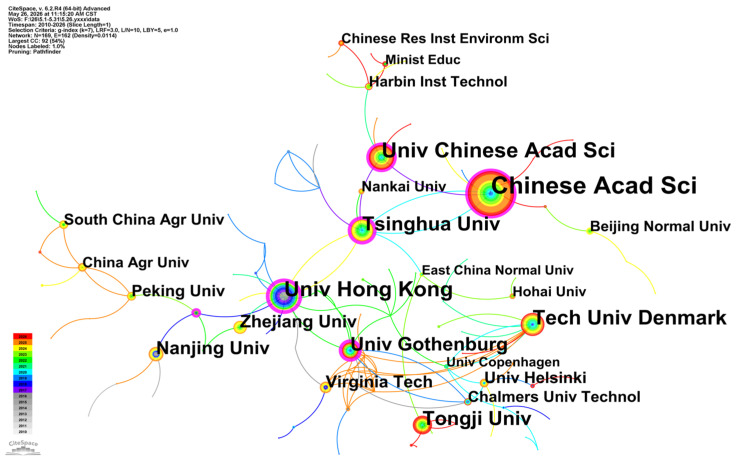
Institutional collaboration network in research on metagenomic surveillance of antimicrobial resistance and pathogenic microorganisms in wastewater systems (2010–2026). Node size is proportional to publication frequency; node colors reflect the year of first appearance. Purple rings indicate betweenness centrality greater than 0.1. Links represent co-authorship relationships between institutions. The network comprises 169 nodes and 162 links (density = 0.0114; largest connected component = 92 nodes, 54%).

The institutional collaboration network comprised 169 nodes and 162 links, with a network density of only 0.0114 (Figure 4). The largest connected component contained only 92 nodes, accounting for 54% of all nodes, indicating that nearly half of the participating institutions were relatively isolated or operated within small disconnected clusters.

Ten of the top 15 institutions were from China, collectively accounting for 73.5% of the total output among the top 15 institutions. The Chinese Academy of Sciences ranked first with 69 publications, followed by the University of Hong Kong with 43, the University of Chinese Academy of Sciences with 39, the Technical University of Denmark with 35, and Tsinghua University with 33. Non-Chinese institutions among the top 15 included the Technical University of Denmark, the University of Gothenburg with 27 publications, the University of Helsinki with 15, Chalmers University of Technology with 13, and Virginia Tech with 17.

The University of Hong Kong showed the highest betweenness centrality among all institutions (0.40), indicating its prominent bridging role in connecting international collaborators. Tsinghua University (0.32) and the University of Gothenburg (0.22) also exhibited substantial intermediary roles. In the network visualization, Chinese institutions formed a dense domestic cluster, with the University of Hong Kong and Tsinghua University serving as the main nodes linking this cluster to international partners.

### 3.4. Author Collaboration Network Analysis

**Figure 5:**
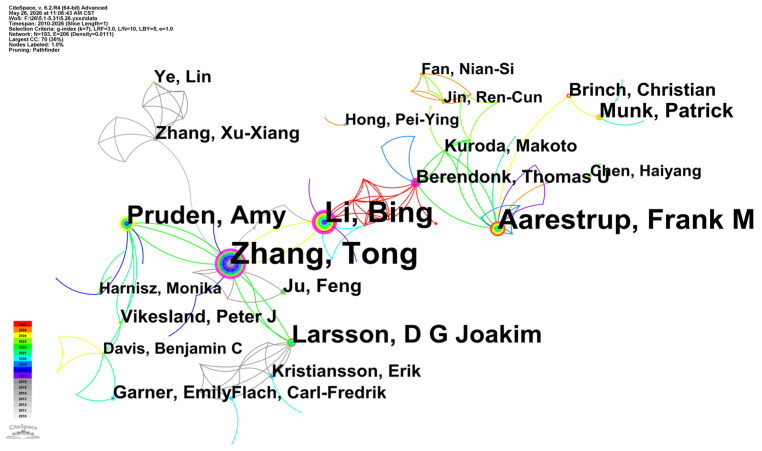
Author collaboration network in research on metagenomic surveillance of antimicrobial resistance and pathogenic microorganisms in wastewater systems (2010–2026). Node size is proportional to publication frequency; node colors reflect the year of first appearance. Links represent co-authorship relationships between individual researchers. The network comprises 193 nodes and 200 links (density = 0.0111; largest connected component = 70 nodes, 36%).

As shown in Figure 5, the author collaboration network contained 193 nodes and 200 links, with a density of 0.0111. The largest connected component included only 70 nodes, accounting for 36% of all nodes, indicating a highly fragmented collaboration structure.

Tong Zhang had the highest publication output, with 38 publications and a betweenness centrality of 0.17, followed by Bing Li with 30 publications and a centrality of 0.14, Frank M. Aarestrup with 26 publications and a centrality of 0.07, Amy Pruden with 20 publications and a centrality of 0.07, and D. G. Joakim Larsson with 17 publications and a centrality of 0.05. The network showed a clear pattern of team-based clustering, with sparse connections between research groups.

Network connectivity decreased progressively from the country level, where all nodes were connected, to the institutional level, where the largest connected component accounted for 54% of nodes, and further to the author level, where it accounted for only 36% of nodes. The implications of this structural pattern are further examined in Section 4.

### 3.5. Subject Category Co-Occurrence Analysis

**Figure 6:**
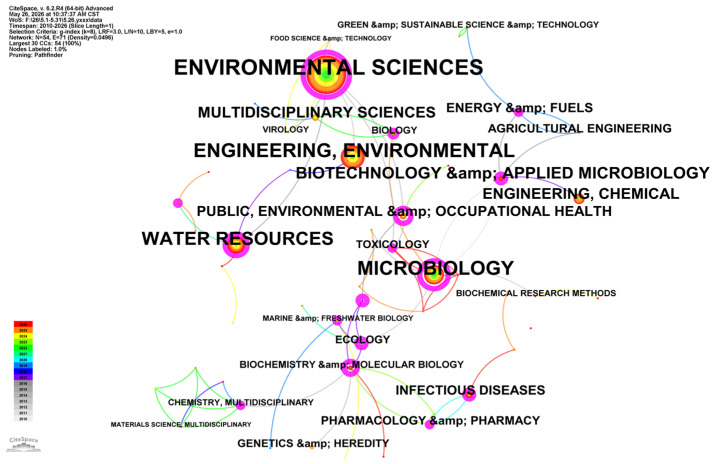
Co-occurrence network of Web of Science categories in research on metagenomic surveillance of antimicrobial resistance and pathogenic microorganisms in wastewater systems (2010–2026). Node size is proportional to publication frequency within each category; node colors reflect the year of first appearance. Purple rings indicate betweenness centrality greater than 0.1. Links represent co-occurrence relationships between categories assigned to the same publication. The network comprises 54 nodes and 71 links (density = 0.0496; largest connected component = 54 nodes, 100%).

The subject category co-occurrence network consisted of 54 nodes and 71 links, with a density of 0.0496, and all nodes were connected (Figure 6).

Environmental Sciences had the highest frequency, with 623 publications, followed by Engineering, Environmental with 330, Microbiology with 247, Water Resources with 187, and Biotechnology and Applied Microbiology with 94.

Betweenness centrality showed a clear asymmetry with frequency. Ecology had the highest betweenness centrality (0.78) but a frequency of only 21, followed by Microbiology (0.70, 247 publications), Biochemistry and Molecular Biology (0.69, 14 publications), and Public Health (0.65, 54 publications). Although Engineering, Environmental ranked second in frequency with 330 publications, its betweenness centrality was only 0.03. The temporal sequence of subject category emergence, from Microbiology and Biotechnology in 2010 to Environmental Sciences in 2013 and then to Public Health and Infectious Diseases in 2014 to 2015, reflects the gradual expansion of the field from its microbiological origins toward a multidisciplinary research domain.

### 3.6. Keyword Co-Occurrence, Clustering, and Timeline Analysis

**Figure 7:**
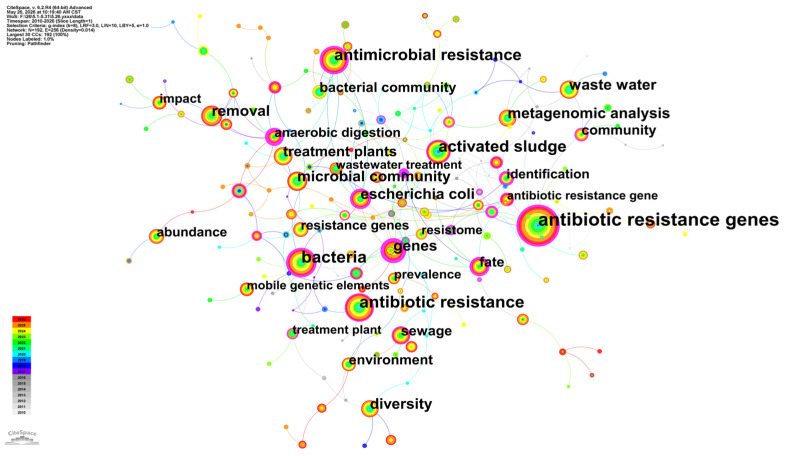
Keyword co-occurrence network in research on metagenomic surveillance of antimicrobial resistance and pathogenic microorganisms in wastewater systems (2010–2026). Nodes represent author keywords (DE field); node size is proportional to co-occurrence frequency. The network comprises 192 nodes and 256 links (density = 0.014).

As shown in Figure 7, the keyword co-occurrence network consisted of 192 nodes and 256 links, with a density of 0.014. The most frequent keyword was “antibiotic resistance genes” with 368 occurrences and a betweenness centrality of 0.28, followed by “antibiotic resistance” with 238 occurrences and a centrality of 0.18, “bacteria” with 220 occurrences and a centrality of 0.26, “antimicrobial resistance” with 177 occurrences and a centrality of 0.28, and “activated sludge” with 166 occurrences and a centrality of 0.12. The keyword “genes” had the highest betweenness centrality, with a value of 0.54.

**Figure 8:**
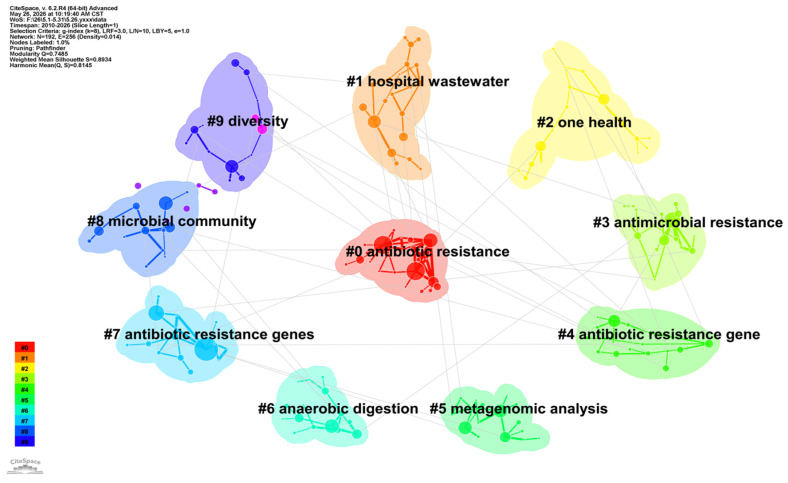
Keyword co-occurrence network with cluster visualization in research on metagenomic surveillance of antimicrobial resistance and pathogenic microorganisms in wastewater systems (2010–2026). Clusters were identified using the log-likelihood ratio (LLR) algorithm and are delineated by colored regions: #0 antibiotic resistance (red), #1 hospital wastewater (orange), #2 one health (yellow), #3 antimicrobial resistance (green), #4 antibiotic resistance gene (light green), #5 metagenomic analysis (cyan), #6 anaerobic digestion (teal), #7 antibiotic resistance genes (light blue), #8 microbial community (blue), and #9 diversity (purple). LLR labels are descriptive and may be semantically overlapping. Accordingly, Clusters #4 and #7 are interpreted jointly as ARG-related clusters rather than as two independent research directions. Network parameters are identical to Figure 7 (N = 192, E = 256; modularity Q = 0.7485; weighted mean silhouette S = 0.8934).

The LLR algorithm identified 10 clusters (Figure 8). The modularity Q value was 0.7485, and the weighted mean silhouette S value was 0.8934, indicating a well-defined and reliable clustering structure [27,28]. These clusters were #0 antibiotic resistance (23 nodes, silhouette = 0.959, mean year = 2016), #1 hospital wastewater (20, 0.898, 2019), #2 one health (19, 0.892, 2020), #3 antimicrobial resistance (18, 0.927, 2018), #4 antibiotic resistance gene (17, 0.687, 2020), #5 metagenomic analysis (16, 0.891, 2016), #6 anaerobic digestion (15, 0.981, 2018), #7 antibiotic resistance genes (15, 1.000, 2015), #8 microbial community (13, 0.902, 2019), and #9 diversity (13, 0.909, 2018). Because AMR-related terminology was highly concentrated in the corpus, several clusters were assigned closely related LLR labels; for example, clusters #0, #3, #4, and #7 were all associated with antibiotic resistance genes. Therefore, in Section 4.4, these 10 clusters are interpreted as five broader research directions.

Clusters #4 and #7 received semantically overlapping ARG-related labels. Because LLR labels are assigned after clustering and serve as descriptive rather than unique thematic definitions, the similarity of these labels should not be interpreted as evidence of two independent research topics. The two clusters differed in size and temporal profile and are therefore interpreted jointly within the broader ARG-related research direction in Section 4.4.

**Figure 9:**
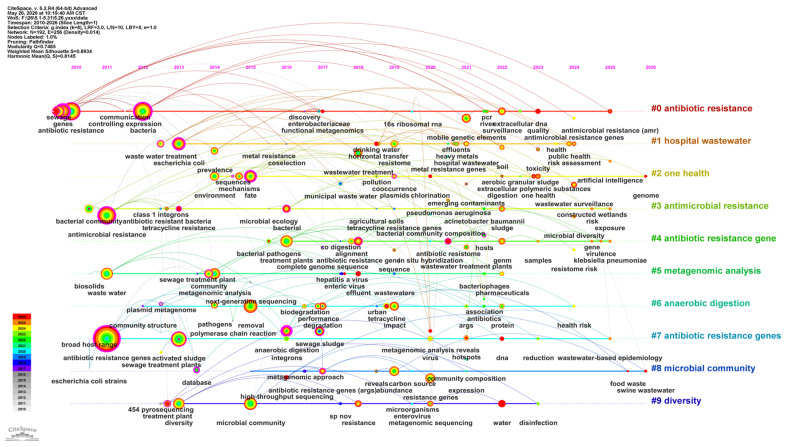
Timeline visualization of keyword clusters in research on metagenomic surveillance of antimicrobial resistance and pathogenic microorganisms in wastewater systems (2010–2026). The horizontal axis represents publication year; each row corresponds to one keyword cluster (labeled on the right). Nodes represent individual keywords, positioned according to their year of first appearance within the dataset. Node size is proportional to co-occurrence frequency; node colors reflect the year of appearance. Connecting lines indicate co-occurrence relationships between keywords within and across clusters.

The keyword timeline (Figure 9) showed a gradual broadening of research themes.

Early keywords from 2010 to 2014 were mainly focused on ARG detection. Between 2015 and 2019, dissemination mechanisms and advanced methodologies became increasingly prominent. After 2020, terms such as “One Health”, “wastewater surveillance”, “public health”, “risk assessment”, “wastewater-based epidemiology”, and “artificial intelligence” emerged during the 2022 to 2025 period.

### 3.7. Keyword Burst Detection

**Figure 10:**
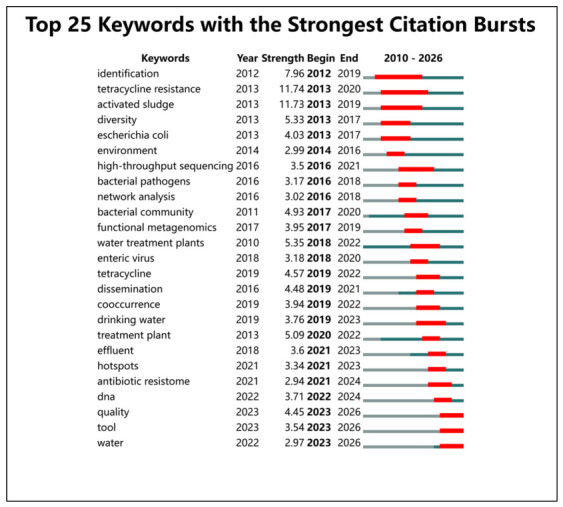
Top 25 keywords with the strongest citation bursts in research on metagenomic surveillance of antimicrobial resistance and pathogenic microorganisms in wastewater systems (2010–2026). Keywords are ranked by the beginning year of the burst period. The “Strength” column indicates burst intensity calculated using the Kleinberg algorithm. The “Begin” and “End” columns denote the start and end years of each burst period. Red segments on the timeline bars indicate years during which a keyword was in a burst state; blue segments indicate non-burst years. Keywords with bursts extending to 2026 (quality, tool, water) represent topics with ongoing heightened research attention at the time of data retrieval.

Burst detection identified 25 keywords (Figure 10). The strongest bursts were observed for “tetracycline resistance” (burst strength = 11.74, 2013 to 2020) and “activated sludge” (11.73, 2013 to 2019).

Only three keyword bursts continued through 2026: “quality” (4.45, 2023 to 2026), “tool” (3.54, 2023 to 2026), and “water” (2.97, 2023 to 2026). “One Health”, “wastewater surveillance”, and “wastewater-based epidemiology” did not appear among the top 25 burst keywords, possibly reflecting gradual rather than abrupt increases in their frequencies [25].

### 3.8. Author Co-Citation Analysis

**Figure 11:**
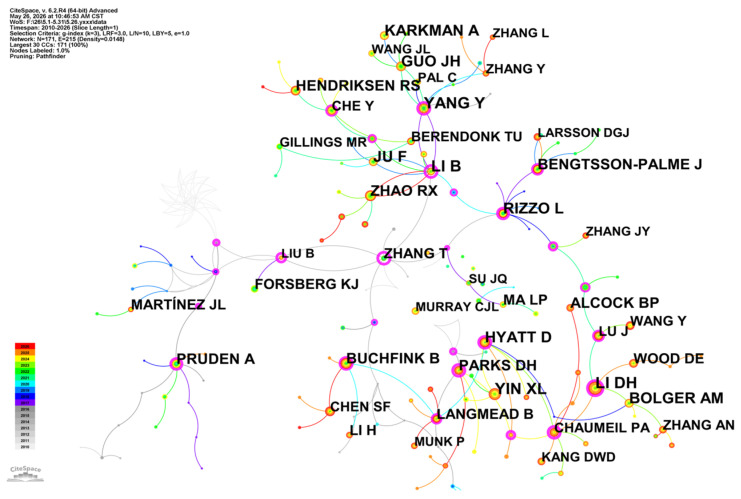
Author co-citation network in research on metagenomic surveillance of antimicrobial resistance and pathogenic microorganisms in wastewater systems (2010–2026). Each node represents a cited author; node size is proportional to co-citation frequency, and purple rings indicate betweenness centrality greater than 0.1. Links represent co-citation relationships (i.e., two authors frequently cited together by articles within the analyzed field). Node colors reflect the year of first co-citation appearance. The network comprises 171 nodes and 215 links (density = 0.0148).

The author co-citation network consisted of 171 nodes and 215 links, with a density of 0.0148, and all nodes were connected (Figure 11). Author co-citation analysis identifies scholars whose work is frequently cited together by publications in the field, thereby revealing the knowledge base on which the research area is built [29].

Yang Y had the highest co-citation frequency, with 244 co-citations, followed by Li B (227), Yin XL (216), Li DH (211), and Ju F (197). A substantial proportion of the top 20 co-cited authors were highly co-cited mainly because of their contributions to widely used bioinformatic resources, rather than wastewater-specific or AMR-focused studies. These authors included Hyatt D, associated with Prodigal; Parks DH, associated with CheckM; Bolger AM, associated with Trimmomatic; Buchfink B, associated with DIAMOND; and Alcock BP, associated with CARD. Yin XL, associated with ARGs-OAP, Wood DE, associated with Kraken, and Li H, associated with BWA and SAMtools, also belonged to this category. The prominence of bioinformatic resource developers among the most frequently co-cited authors underscores the essential role of computational infrastructure in wastewater metagenomics.

### 3.9. Journal Co-Citation Analysis

**Figure 12:**
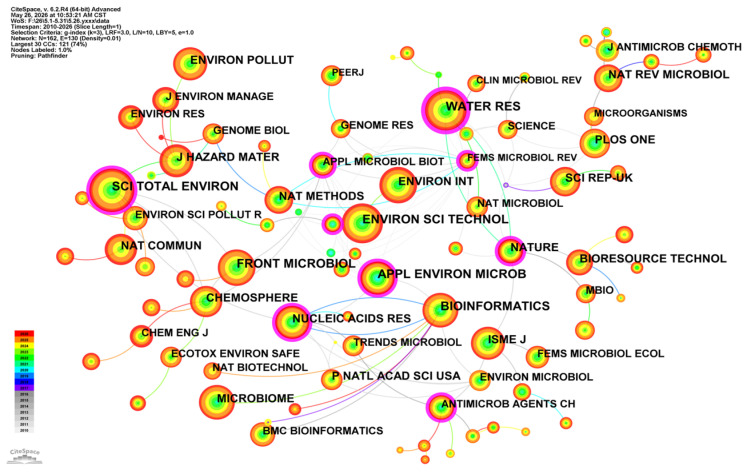
Journal co-citation network in research on metagenomic surveillance of antimicrobial resistance and pathogenic microorganisms in wastewater systems (2010–2026). Each node represents a co-cited journal; node size is proportional to co-citation frequency. Purple rings indicate betweenness centrality greater than 0.1. Links represent co-citation relationships between journals. The network comprises 162 nodes and 130 links (density = 0.01; largest connected component = 121 nodes, 74%).

As shown in Figure 12, the journal co-citation network comprised 162 nodes and 130 links, with the largest connected component consisting of 121 nodes (74% of the network).

*Water Research* ranked first in co-citation frequency (977), followed by *Science of the Total Environment* (946), *Environmental Science & Technology* (864), *Frontiers in Microbiology* (762), and *Applied and Environmental Microbiology* (683). The co-cited journals were grouped into three major clusters corresponding to environmental science and engineering, microbiology, and bioinformatics. *Water Research* exhibited the highest betweenness centrality (0.25), indicating its pivotal role as a knowledge bridge connecting different disciplinary clusters within the network.

### 3.10. Literature Co-Citation Analysis

**Figure 13:**
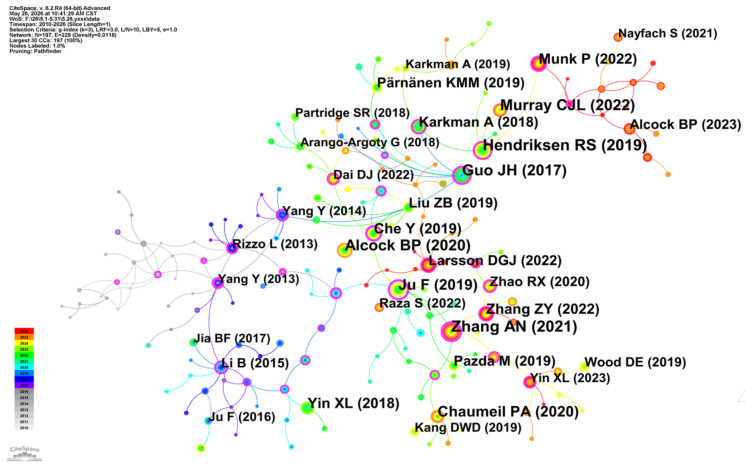
Reference co-citation network in research on metagenomic surveillance of antimicrobial resistance and pathogenic microorganisms in wastewater systems (2010–2026). Each node represents a co-cited reference, labeled by first author and publication year. Node size is proportional to co-citation frequency; purple rings indicate betweenness centrality greater than 0.1. Node colors reflect the publication year, ranging from cool tones (earlier) to warm tones (recent). The network comprises 197 nodes and 228 links (density = 0.0118; largest connected component = 197, 100%).

The reference co-citation network comprised 197 nodes and 228 links, forming a fully connected network (Figure 13).

The most frequently co-cited reference was Zhang et al. (2021) [15], published in *Nature Communications* (99 co-citations, betweenness centrality = 0.45), followed by Hendriksen et al. (2019) [14] (95 co-citations, centrality = 0.30) and Guo et al. (2017) [7] in *Water Research* (87 co-citations, centrality = 0.52). Ju et al. (2019) [30], published in *The ISME Journal*, exhibited the highest betweenness centrality in the network (0.84), indicating that this reference served as a critical intellectual bridge connecting multiple thematic clusters within the network. By contrast, Murray et al. (2022) [1] in *The Lancet* (79 co-citations) had a betweenness centrality of zero, suggesting that the public-health perspective has not yet been fully integrated with the technical surveillance literature. Foundational references in this domain included Rizzo et al. (2013), Yang et al. (2014), and Li et al. (2015) [31,32,33].

The four-tier knowledge structure revealed by the co-citation analysis, encompassing environmental microbiology, wastewater engineering, global surveillance, and public health, was consistent with the findings from the discipline co-occurrence analysis (Section 3.5) and the keyword timeline analysis (Section 3.6).

## 4. Discussion

### 4.1. The Development Trajectory of the Field

Annual publication output remained relatively limited during the early years, increased after 2018, and accelerated further after 2020, as described in Section 3.1. These periods were defined descriptively to summarize the publication trajectory and are not statistically estimated structural breaks.

The expansion of this field likely reflects several concurrent developments. Advances in high-throughput sequencing have progressively increased the accessibility and analytical capacity of metagenomic research [34]. At the same time, the maturation of resistome databases, computational classifiers, and metagenomic analytical pipelines has enabled more comprehensive characterization of antimicrobial resistance determinants in complex wastewater communities [7,35,36]. Increasing international policy attention to environmental antimicrobial resistance may also have contributed to sustained research activity [37].

The post-2020 increase coincided with the rapid expansion of wastewater-based epidemiology during the COVID-19 pandemic [18]. Sampling networks, laboratory workflows, and institutional experience developed for SARS-CoV-2 monitoring may provide a foundation for subsequent AMR and pathogen surveillance [38,39]. However, the bibliometric data cannot establish that the pandemic caused the observed growth. Wastewater surveillance and enteric-virus monitoring had already developed before 2020, suggesting that the pandemic may have accelerated an existing research trajectory rather than initiated an entirely new direction.

The findings of this study can be contextualized through comparison with several existing bibliometric analyses. Nizeyimana et al. [20] analyzed 176 publications on the impact of WWTP effluents on ARG profiles in receiving water bodies (1990–2023). Despite differences in scope, several findings converge: both studies identified China as the dominant contributor with relatively low betweenness centrality, fragmented collaboration networks, and One Health as an emerging framework. The present study further documents the post-2020 acceleration in growth and the emergence of WBE. Hui et al. [21] analyzed 7925 publications on emerging contaminants in groundwater (1999–2024), employing a more diverse set of quantitative indicators including growth rates and trend factors. Both studies observed an acceleration around 2017–2018 accompanied by increasing interdisciplinarity. The parallel acceleration across different environmental AMR domains suggests that the trends reported in the present study are part of a wider expansion of environmental resistance research. Compared with the bibliometric analysis by Garrido-Cardenas et al. [24], which covered wastewater metagenomics up to 2016, the present analysis captures a substantially different landscape. None of the ten keyword clusters delineated in this study were present as independent thematic clusters prior to 2016.

### 4.2. Global Research Landscape and Collaboration Models

Collaboration network analyses spanning the country, institution, and author levels (Section 3.2, Section 3.3 and Section 3.4) revealed a research landscape characterized by three structural features: high concentration of output in a few countries and institutions, moderately productive actors taking on the role of connecting different groups, and fragmentation of author collaboration. Similar patterns have been reported in bibliometric analyses of microplastics in aquatic environments and in the broader environmental AMR literature [22,26]. These features were consistent across the three analytical scales and carry distinct implications for the development of the field.

The first feature of this landscape is concentrated output with limited international bridging. The dominant position of China (approximately 49% of total output) reflects substantial national investment in environmental AMR research, driven by high antimicrobial consumption rates and extensive wastewater treatment infrastructure [34]. However, China exhibited low betweenness centrality at the national level, indicating that its relatively limited bridging role in connecting different cooperation groups among countries [40]. The United States, the second-largest contributor, displayed similarly low betweenness centrality, indicating that the pattern of high output coupled with low bridging is not unique to China. Together, the two countries accounted for approximately 68% of total output yet contributed minimally to the structural connectivity of the network.

The second feature is that several countries with moderate output served critical intermediary functions. These countries had moderate publication volumes but high betweenness centrality, playing a disproportionately large role in facilitating knowledge exchange between otherwise loosely connected research communities. Australia, Sweden, Spain, India, Brazil, and Finland served as the principal nodes with high centrality in the network. Several of these countries maintained simultaneous collaborative ties with the major producers (China and the United States) and with broader European or Global South research communities, enabling them to function as structural intermediaries. India is notable both as a major research contributor and as a country bearing a high AMR burden [41], demonstrating that meaningful research engagement is achievable for regions facing severe resistance challenges. The brokerage role of India likely reflects its concurrent collaborative linkages with Western research institutions and with other countries across Asia and Africa [42,43].

The third feature manifested at the institutional level, where concentration was more pronounced and a small number of institutions served key intermediary roles. The dominance of Chinese institutions was particularly pronounced. The largest connected component contained only approximately half of all nodes, indicating that nearly half of the participating institutions remained relatively isolated. Within this landscape, the University of Hong Kong occupied a structurally distinctive position, exhibiting the highest institutional betweenness centrality. This brokerage role reflects its interface position between the mainland Chinese research system and the international academic community, enabling it to serve as a node linking Chinese domestic research with European and North American partners. A comparable yet weaker bridging function was served by Tsinghua University. Outside China, the University of Gothenburg and the Technical University of Denmark functioned as principal European bridging nodes; the Technical University of Denmark was the most productive European institution. Virginia Tech was the only US institution among the top 15, suggesting that US contributions to this field are distributed across many institutions rather than concentrated in a few.

Among the three levels, fragmentation was most pronounced in the author collaboration network, with direct implications for methodological standardization. The largest connected component encompassed only approximately one-third of all nodes. The five major clusters identified in Section 3.4, centered on Zhang Tong (University of Hong Kong), Aarestrup (Technical University of Denmark), Pruden (Virginia Tech), Larsson (University of Gothenburg), and Berendonk (Technische Universität Dresden), represent mature research teams with distinct thematic and geographic identities. The Zhang Tong cluster focuses on wastewater resistome ecology and metagenomic methodology [30]; the Aarestrup cluster on large-scale global wastewater AMR and pathogen surveillance [10]; the Pruden cluster on ARG dissemination in water infrastructure systems [44]; the Larsson cluster on the molecular ecology of environmental AMR [2]; and the Berendonk cluster on European freshwater AMR monitoring. Collectively, these clusters cover a broad thematic scope, yet the links between them are sparse, meaning that methodological protocols, analytical pipelines, and data reporting standards have largely evolved in parallel rather than through coordinated harmonization.

This fragmentation carries direct practical consequences. The keyword bursts for “quality” and “tool” persisting through 2026 (Section 3.7) suggest that the field is beginning to recognize the need for analytical standardization; however, the weak collaborative ties among the teams best positioned to lead this effort may impede progress. In the absence of direct collaboration or joint benchmarking exercises between major clusters, the risk of methodological incompatibility will persist, potentially limiting the comparability of surveillance data across studies and regions.

Africa, the Middle East, and Central Asia were weakly represented in the indexed corpus. This underrepresentation is particularly noteworthy given that these regions bear the highest AMR-attributable mortality rates globally [19], while simultaneously facing limited wastewater treatment infrastructure and weak antimicrobial stewardship regulatory capacity. The presence of South Africa in the network demonstrates that meaningful research engagement in an African context is achievable with targeted institutional investment and international collaborative support [38,39]; however, this remains an isolated case rather than a regional trend. It is important to acknowledge that this geographic disparity may be partially attributable to database coverage biases, which are addressed further in the limitations section. Bridging this gap will require targeted strategies, including technology transfer programs that provide sequencing infrastructure, the development of low-cost sampling and analytical protocols adapted to resource-limited settings, and the systematic inclusion of low-income country partners in global wastewater surveillance consortia [10,14,20].

### 4.3. Interdisciplinary Structure in This Field

#### 4.3.1. Disciplinary Diversity and Comparative Context

The discipline co-occurrence (Section 3.5) and journal co-citation (Section 3.9) analyses provided converging evidence that this field operates at the intersection of multiple disciplinary domains. This co-occurrence network contains 54 WoS subject classifications, spanning multiple fields such as environmental science, life science, engineering technology, and public health. Most disciplines are connected to each other, forming a connected body with environmental science and microbiology as the core. This shows that this field has a high degree of interdisciplinary research. In recent years, computing disciplines such as mathematics and computational biology, and information systems have also emerged, but the frequency of occurrence is very low and they are still at the edge of the network. Compared with other bibliometric analyses, the present study identified a higher degree of disciplinary diversity. Sweileh and Moh’d Mansour [26], in a bibliometric analysis of 2611 publications on environmental AMR research broadly defined (2000–2019), concluded that the environmental component of AMR research is the most dynamic yet most neglected dimension among the three pillars of One Health. Their analysis covered soil, water, air, and food-related matrices but identified a narrower disciplinary distribution than the subject categories observed in the present study.

The broader disciplinary coverage documented in this study likely reflects two distinctive features of wastewater metagenomic surveillance. First, the adoption of metagenomics as the core methodology introduces computational and bioinformatics categories that are absent from culture-based or targeted gene studies [45,46]. Second, pathogen monitoring has been more clearly included in the scope of research, allowing disciplines such as public health and infectious diseases to be more involved, which is not prominent in ARG research that focuses on treatment processes [1,14].

#### 4.3.2. Pillar and Bridging Roles of Individual Disciplines

Different disciplines assumed distinct pillar and bridging roles within the network. Environmental Sciences was the dominant disciplinary affiliation by publication volume and maintained high betweenness centrality, serving both as the primary outlet for research output and as a key intermediary connecting other disciplines. This dominance was mirrored in the journal co-citation network, where the three most frequently co-cited journals, *Water Research*, *Science of the Total Environment*, and *Environmental Science & Technology*, all belong to the environmental science and engineering domain. *Water Research* exhibited the highest betweenness centrality in the co-citation network, indicating that it serves as the principal conduit for knowledge flow between the environmental engineering and microbiology subcommunities.

Microbiology was the earliest-appearing category (2010), entering the network simultaneously with Biotechnology & Applied Microbiology, and exhibited the second-highest betweenness centrality in the co-occurrence network. This dual characteristic of earliest emergence and strong bridging capacity reflects the foundational status of Microbiology, given that metagenomics is fundamentally a microbiological methodology and antimicrobial resistance is inherently a microbiological phenomenon [47,48]. In the journal co-citation network, microbiology journals including *Frontiers in Microbiology*, *Applied and Environmental Microbiology*, and *The ISME Journal* formed a distinct cluster, connected to the environmental cluster primarily through *Water Research* and *Applied and Environmental Microbiology* [2]. Another key structural feature is the strong bridging roles assumed by several disciplines with relatively low publication frequencies. Ecology had the highest betweenness centrality of all categories (0.78), despite a publication volume far below that of many technical disciplines. This bridging role may reflect the adoption of ecological concepts such as community assembly theory, diversity metrics, and niche differentiation in wastewater microbiome research [4,30,49], linking environmental monitoring studies with fundamental ecological theory. Biochemistry & Molecular Biology provided mechanistic underpinnings for understanding horizontal gene transfer and mobile genetic elements [8], bridging descriptive surveillance studies and mechanistic molecular investigations. Public, Environmental & Occupational Health connected the environmental and clinical dimensions, translating wastewater surveillance data into risk assessment frameworks. These three disciplines are not major sources of publications in this field but rather serve as critical conduits for knowledge flow between otherwise separated research communities [25].

This pattern contrasts sharply with Engineering, Environmental, which exhibited the opposite profile: high publication frequency but extremely low bridging function. This suggests that engineering-oriented research focused on ARG removal through wastewater treatment technologies has largely developed within its own disciplinary boundaries, with limited knowledge exchange with the microbiological, ecological, or public-health components of the field. A practical consequence of this relative disciplinary insularity is a potential disconnect between treatment technology development and surveillance-informed intervention design. Treatment research may optimize ARG removal without reference to surveillance data that could identify which resistance determinants pose the greatest public-health risks, while surveillance studies may document ARG occurrence without informing the engineering solutions needed for mitigation.

#### 4.3.3. Temporal Formation of the Interdisciplinary Structure

The year of first appearance of each discipline in the co-occurrence network traces the formation pathway of the field’s interdisciplinary structure. Microbiology and Biotechnology & Applied Microbiology appeared first (2010), reflecting the origins of culture-independent microbiological analysis. Environmental Sciences and Ecology joined in 2013, marking an expansion of the research agenda from microbiological characterization to ecosystem-scale environmental monitoring. Public, Environmental & Occupational Health entered in 2014, followed by Infectious Diseases in 2015, marking the initial engagement of public health and clinical perspectives. Water Resources also appeared in 2015, followed by Engineering, Chemical in 2017. This temporal sequence, progressing from microbiology through environmental science to public health and engineering, describes a pattern of progressive expansion outward from a microbiological core. Notably, Veterinary Sciences had only minimal participation, indicating that the animal health dimension of the One Health framework has not yet been substantively incorporated into this research field, a gap discussed further in Section 4.5.

#### 4.3.4. Computational Infrastructure as a Fifth Dimension

Beyond the features discussed above, computational infrastructure constitutes a fifth structural dimension of this field. The author co-citation analysis (Section 3.8) revealed that a substantial proportion of the top 20 co-cited authors are developers of bioinformatics tools. The journal co-citation network further corroborated this finding, with *Bioinformatics* and *Nucleic Acids Research* ranking among the top 10 co-cited journals. The high betweenness centrality of *Nucleic Acids Research* reflects its role as the primary publication venue for reference databases such as CARD and NCBI resources, which are cited across all disciplinary subcommunities. Together, these observations indicate that computational science constitutes a fifth structural dimension of the field, one not encompassed by the conventional four research domains (environmental science, microbiology, engineering, and public health) yet indispensable to its development. The field’s reliance on external computational infrastructure is noteworthy. Wastewater metagenomic monitoring is largely built on general and specialized bioinformatics tools and databases [50,51], so the update and maintenance status of these tools and databases may affect the analytical capabilities of future monitoring.

### 4.4. Core Research Topics in This Field

The keyword clusters identified in Section 3.6 were interpreted at a broader conceptual level to avoid overemphasizing differences among semantically overlapping labels. These clusters can be synthesized into five major research directions, which collectively describe the evolution of wastewater metagenomic research from environmental characterization toward broader surveillance and public-health applications.

The first direction concerns the detection and characterization of antibiotic resistance determinants in wastewater systems. This foundational theme is represented primarily by ARG-related clusters, including Cluster #0 and Cluster #7. Although some ARG-related labels showed lexical overlap, they collectively reflect the early development of wastewater resistome research, particularly the identification and distribution of resistance genes in wastewater treatment systems. Cluster #7 is the earliest-forming thematic group in the network, reflecting the field’s origins in ARG surveys within activated sludge and sewage treatment processes. The reference co-citation analysis (Section 3.10) identified corresponding foundational works, including Yang et al. 2014 [33], Li et al. 2015 [31], and Guo et al. 2017 [7], which established the early framework for applying metagenomic approaches to wastewater resistome analysis. The continued activity of Clusters #0 and #7 through 2026 on the timeline map (Figure 9) indicates that ARG characterization remains a sustained research activity even as the field broadens, providing the descriptive foundation for all subsequent directions. Recent studies continue to extend this descriptive foundation; for example, municipal-scale shotgun metagenomics across Latvian cities resolved geographically structured urban resistome profiles shaped by local industrial and healthcare inputs [52].

The second direction addresses the fate of ARGs during wastewater treatment. Clusters #6 and #9 represent the engineering dimension of the field, investigating whether and how treatment processes reduce ARG loads in wastewater and sludge. Cluster-defining terms include anaerobic digestion, activated carbon, constructed wetlands, and pharmaceutical wastewater, reflecting the evaluation of diverse treatment technologies for ARG removal. The presence of “454 pyrosequencing” in Cluster #9 is noteworthy as a marker of early sequencing technology, confirming that this direction was already established before the widespread adoption of shotgun metagenomics. This direction is directly linked to the discipline co-occurrence finding in Section 3.5 that Engineering, Environmental had high publication frequency but extremely low betweenness centrality. The combination of active treatment-oriented research themes with disciplinary insularity suggests that engineering research directed toward ARGs removal has developed somewhat independently of the microbiological and public-health components of the field, representing an opportunity for enhanced cross-disciplinary integration.

The third direction involves the monitoring of wastewater from specific high-risk sources. Clusters #1 and #3 mark a shift from general wastewater studies to targeted monitoring of specific high-risk sources. Hospital wastewater has received particular attention because it concentrates antimicrobial residues, multidrug-resistant bacteria, and patients undergoing antimicrobial therapy, conditions that select for and amplify resistance [34], and gene flow is frequently observed in sewage from different buildings of hospital [53]. Most ARGs could not be significantly removed by chlorination treatment in the hospital wastewater treatment system [54]. The inclusion of livestock-related terms in Cluster #3 extends the surveillance scope to agricultural sources. Cluster #8 adds a further dimension to this direction by documenting the co-occurrence of ARGs and heavy metal resistance genes in sludge, pointing to co-selection mechanisms whereby heavy metal contamination may indirectly promote antibiotic resistance even in the absence of direct antimicrobial pressure [55,56]. This co-selection phenomenon carries broader implications for treatment design, as it suggests that effective AMR mitigation may require simultaneously addressing antimicrobial residues and co-selective agents such as heavy metals and biocides [57,58,59].

The fourth direction encompasses methodological expansion and broadening of the analytical scope. Clusters #5, together with related ARG- and pathogen-associated clusters, represent the evolving methodological toolkit of the field. The defining terms of these clusters include viral metagenomics, virulence factors, bacteriophages, and VRE (vancomycin-resistant enterococci), indicating a clear expansion of the analytical scope beyond bacterial resistance genes. The inclusion of viral metagenomics reflects a growing recognition that bacteriophages serve as vehicles for horizontal ARG transfer, and that viral communities in wastewater carry functional information relevant to pathogen surveillance [60,61,62]. The co-occurrence of “virulence factors” alongside resistance gene terms marks an important conceptual shift: from merely monitoring whether ARGs are present in wastewater to asking whether those ARGs are carried by microorganisms with pathogenic potential. Recent comparative-genomic work illustrates this pathogen-centered shift, showing that carbapenemase-producing Enterobacterales recovered from wastewater can be genetically near-identical to isolates from national clinical surveillance, including *Escherichia coli* and *Klebsiella pneumoniae* that share highly homologous carbapenemase-carrying plasmids across wastewater and clinical sources [63].This expansion is underpinned by the computational infrastructure documented in the co-citation analysis (Section 3.10), in which the CARD database [45] and the ARGs-OAP analytical pipeline [51] have become foundational to the field’s methodological capacity.

The fifth direction concerns One Health integration and public-health orientation. Cluster #2 is the latest-forming thematic group, encompassing wastewater surveillance, emerging contaminants, and the One Health framework. The keyword data reveal a progressive expansion of risk characterization scope across the five directions: from individual ARGs in activated sludge, through the resistome concept as an integrated analytical unit, to virulence factors and specific clinically relevant pathogens such as *Klebsiella pneumoniae* and VRE. This progression represents a significant shift in the field’s risk assessment framework, from documenting the environmental presence of resistance determinants toward evaluating their clinical relevance, an evolution consistent with the broader transformation from environmental characterization to public-health surveillance discussed in detail in Section 4.5 [64,65,66].

The alignment between the five research directions identified through data-driven keyword clustering and the priority areas recognized by international policy frameworks provides an external reference point for these findings. The United Nations Environment Programme report *Bracing for Superbugs* [67] identified three sectors requiring priority environmental interventions for AMR containment: wastewater and sewage management, pharmaceutical manufacturing, and agricultural production. The first and third of these correspond directly to the thematic clusters documented in this study: Clusters #1 and #6 address wastewater-related issues, while Cluster #3 incorporates livestock sources. This comparison reveals a notable gap: pharmaceutical manufacturing wastewater does not appear as a distinct research theme in the keyword network, despite the UNEP report emphasizing it as a major point source of AMR selection pressure. This omission may partly reflect the geographic concentration of pharmaceutical manufacturing, particularly antibiotic API and intermediate production, in a limited number of countries [68,69], resulting in publication volumes below the threshold for cluster formation in this dataset; nevertheless, it highlights a research area where the current literature has not yet matched the urgency articulated by policy.

Because LLR labels are generated algorithmically and may contain lexical redundancy, cluster identities were interpreted based on their overall keyword composition rather than individual labels alone. The keyword clusters should be regarded as computationally derived groupings rather than predefined biological categories. Therefore, semantically overlapping clusters, particularly those related to ARG terminology, were interpreted together at the broader thematic level. This conservative interpretation reduces the influence of keyword-level redundancy on the identification of major research directions.

### 4.5. Emerging Shift in Research Emphasis from Environmental Characterization to Public-Health Surveillance

The bibliometric patterns identified in this study are consistent with a gradual change in research emphasis. Earlier literature focused predominantly on ARG occurrence, activated sludge, treatment processes, and removal efficiency, whereas more recent publications increasingly incorporate wastewater surveillance, One Health, public health, risk assessment, and clinically relevant pathogens.

Similar broadening is visible in the category and co-citation networks, where public-health and infectious-disease perspectives occur alongside the established environmental science and microbiology domains. Wastewater studies have increasingly examined whether detected resistance determinants are mobile, associated with pathogenic hosts, or relevant to population-level surveillance [9,17,49]. These associations indicate increasing conceptual engagement across fields, but category co-occurrence and bibliometric centrality cannot demonstrate that clinical, veterinary, and environmental surveillance systems are operationally connected.

The expansion of wastewater-based epidemiology during the COVID-19 pandemic coincided with the increased visibility of surveillance-oriented research [70,71]. Infrastructure established for SARS-CoV-2 monitoring may provide a foundation for AMR surveillance, including routine sampling networks, nucleic-acid analysis, reporting systems, and communication between laboratories and public-health agencies. Nevertheless, the present analysis cannot establish a causal effect of the pandemic or confirm that such infrastructure has been routinely repurposed for AMR surveillance.

However, public-health-oriented terminology remains less prominent than environmental and treatment-oriented research. The current evidence therefore supports an emerging orientation toward health applications, while indicating that operational integration, prospective validation, and decision-making use remain limited. Direct evidence from surveillance programs, policy records, clinical datasets, and implementation evaluations is required before operational public-health integration can be demonstrated. The keyword burst analysis (Section 3.7) reinforces this assessment: “one health,” “wastewater surveillance,” and “public health” are all absent from the top 25 burst keywords. The growth of these concepts has been gradual rather than explosive, which explains why the Kleinberg algorithm did not flag them, and also implies that they have not yet generated the concentrated attention characteristic of a mature research front [72].

### 4.6. Research Gaps and Future Directions

The bibliometric patterns documented in Section 3.1, Section 3.2, Section 3.3, Section 3.4, Section 3.5, Section 3.6, Section 3.7, Section 3.8, Section 3.9 and Section 3.10 and interpreted in Section 4.1, Section 4.2, Section 4.3, Section 4.4 and Section 4.5 collectively point to the intersection of the field’s current developmental trajectory and identifiable knowledge gaps. These frontiers span three levels: improvements to the methodological infrastructure, the establishment of translational frameworks linking environmental data to health outcomes, and the structural conditions necessary to sustain progress on both.

#### 4.6.1. Methodological Frontiers

At the methodological level, the most pressing priority is analytical standardization. The keyword bursts for “quality” and “tool” both persist through 2026 (Section 3.7). These keywords are themselves broad and the associated interpretation is preliminary; however, their continued presence may indicate that the field is beginning to confront a challenge deferred by rapid growth: the lack of widely accepted standardized protocols for core steps in wastewater metagenomic analysis. Compared with other metagenomic matrices, wastewater presents particular standardization challenges, including high concentrations of PCR inhibitors, extremely high microbial diversity, large inter-sample variability, and the need to capture both chromosomal and extrachromosomal resistance determinants simultaneously [73,74]. Substantive progress will require the development of reference materials matched to wastewater matrices [75], interlaboratory comparisons to quantify variation across laboratories in DNA extraction recovery and bioinformatic taxonomic classification [76], and the progressive adoption by the field of standardized, shared, version-controlled, and fully parameterized analytical pipelines [13]. The fragmented structure of the author collaboration network, in which the five major clusters operate largely independently (Section 3.4), indicates that such harmonization has not yet materialized and will require deliberate multi-team coordination, for example through international collaborative mechanisms modeled on clinical metagenomics benchmarking consortia [77].

The second methodological frontier is the integration of long-read sequencing into surveillance workflows. The keyword analysis documents a progressive shift in analytical targets: from individual ARGs, to whole-resistome profiling, to characterization of mobile genetic elements and virulence factor assessment. This shift toward element-level and strain-level resolution creates technical demands that short-read sequencing cannot fully meet.

Determining whether a detected ARG resides on a transferable plasmid within a pathogenic host requires contiguous sequence assembly spanning tens to hundreds of kilobases. Platforms such as Oxford Nanopore and PacBio are now capable of delivering this capacity from complex metagenomic samples [78]. The portability of nanopore devices offers the possibility of deployment in resource-limited settings where the participation gaps documented in Section 3.2 are most acute [79].

The appearance of “artificial intelligence” within the “one health” keyword cluster (Section 3.6) marks a third methodological frontier. In the specific context of wastewater AMR surveillance, the applications most likely to generate impact are those that address bottlenecks particular to this field, including inferring the transferability and host range of resistance determinants from genomic context, detecting anomalous resistome shifts in longitudinal monitoring data that may precede clinically observable changes, and integrating metagenomic, chemical, and epidemiological data streams into composite risk indicators [21]. Realizing these applications will depend on the accumulation of large, curated wastewater metagenomic datasets; the field is progressing toward the required scale and standardization, but has not yet arrived. Global sewage resistance surveillance has expanded to over one hundred countries [10], while at the same time, harmonized methods for aquatic environmental resistance monitoring are still being developed through consensus frameworks [80].

#### 4.6.2. Translational Priorities

At the translational level, the environment-to-public-health translational gap identified in Section 4.5 points to two research needs. The first is a quantitative risk assessment framework capable of bridging the distance between metagenomic detection and health outcome estimation. Such a framework must address a series of interconnected questions: Is the detected ARG carried by a mobile genetic element with demonstrated transfer capacity? Does the host microorganism carry functional virulence determinants? Through which exposure pathways can wastewater-borne resistance reach human populations? What proportion of the clinical resistance burden is attributable to the wastewater pathway relative to other transmission routes? Existing risk assessment frameworks address individual steps in this process, but no integrated model links metagenomic surveillance outputs to quantitative health risk estimates covering all pathways [2].

The second priority is a transition from cross-sectional characterization to longitudinal surveillance designs. The appearance of “surveillance” and “wastewater-based epidemiology” in the keyword timeline (Section 3.6) reflects a growing recognition of this need. The keyword burst for “water” persisting through 2026 (Section 3.7) is broad in scope but may also indicate an expansion of monitoring coverage from wastewater treatment facilities to broader water environments, including surface water, drinking water sources, and water reuse systems [81,82].

To bridge the translational gap, future research should move beyond cross-sectional detection and adopt prospective, multi-site longitudinal designs that pair wastewater metagenomic measurements with contemporaneous clinical AMR data, antimicrobial consumption records, and veterinary surveillance. Methodological priorities include standardized sampling and bioinformatic workflows, reliable assignment of ARGs to microbial hosts and mobile genetic elements, and quantitative risk models that integrate ARG abundance, mobility, virulence potential, exposure pathways, and health outcomes. Translation into practice will further require researchers and public-health agencies to jointly define alert thresholds, reporting intervals, and response protocols. These approaches should then be evaluated prospectively to determine whether wastewater-derived signals improve the timeliness, targeting, or effectiveness of public-health interventions.

#### 4.6.3. Structural Conditions for Sustained Progress

At the structural level, two systemic conditions will determine the field’s capacity to advance along the frontiers outlined above. The first is globally equitable participation. The geographic gaps documented in Section 3.2, particularly the near-absence of sub-Saharan Africa, the Middle East, and Central Asia from the research landscape, limit the global representativeness of surveillance data and exclude the populations bearing the heaviest AMR burden [19]. Global wastewater surveillance initiatives have demonstrated that multi-country inclusion is operationally feasible [10,14], but extending coverage to underrepresented regions will require technology transfer, simplified field-deployable protocols, and open access data platforms enabling monitoring sites to benchmark their results against international baselines.

The second condition is the operational implementation of One Health surveillance networks. The bibliometric evidence on the current state is unambiguous: Veterinary Sciences contributed only approximately 4 publications to this field (Section 3.5); no scholars from clinical epidemiology or veterinary medicine appear in the author co-citation network (Section 3.8); and the most prominent public health reference in the co-citation network occupies a position of structural isolation (Section 3.10). Moving from conceptual integration to operational implementation will require institutional arrangements that routinely connect wastewater metagenomic surveillance outputs with clinical AMR surveillance databases, veterinary resistance monitoring systems, antimicrobial consumption registries, and public-health decision platforms. Future studies should not only evaluate whether wastewater signals correlate with clinical resistance trends, but also determine how these signals can be incorporated into early-warning systems, intervention prioritization, and resource allocation strategies. The infrastructure established during the COVID-19 pandemic for SARS-CoV-2 wastewater monitoring provides a starting point, but redirecting it toward integrated AMR and pathogen surveillance will require sustained cross-sectoral governance extending well beyond the research community itself [20].

Beyond generating surveillance data, future research should evaluate the implementation performance of wastewater-based AMR surveillance systems. Such evaluations should examine whether metagenomic indicators provide timely information beyond existing clinical surveillance approaches, whether they improve detection of emerging resistance threats, and whether they lead to measurable improvements in public-health responses. Establishing this evidence base will be essential for transforming wastewater metagenomics from a research-oriented monitoring approach into an operational component of One Health surveillance systems.

### 4.7. Limitations

This study has several limitations:

First, the data were drawn exclusively from the WoS CC, which is biased toward English-language international journals and has limited coverage of local journals in Africa and parts of Asia. The exclusion of non-English publications, together with the English-language indexing profile of the WoS CC, may have underrepresented locally published research from some regions. Although only one non-English record was excluded from the retrieved dataset, language restrictions may interact with database coverage and affect geographical representation. Therefore, the geographical patterns reported in this study describe the indexed English-language literature and should not be interpreted as a complete measure of regional research activity or research capacity. The future studies should incorporate Scopus, PubMed, or regional databases for verification.

Second, only CiteSpace was used for the analysis; although the high modularity and silhouette values indicate well-defined clusters with clear inter-cluster differentiation, cross-validation using VOSviewer or Bibliometrix would provide an additional robustness check.

Third, the 2026 data are incomplete, with the search conducted up to 22 May. Records from 2026 were included in co-occurrence and co-citation network construction to preserve the most recent network topology, but excluded from annual publication growth-rate calculations and phase delineation analyses. Consequently, keyword bursts extending into 2026 are partially based on incomplete data, and their persistence awaits confirmation with a complete annual dataset.

Fourth, the keyword analysis relies on author-assigned keywords, which may contain terminological inconsistencies. Although such variations were considered conservatively during interpretation, incomplete keyword harmonization before network construction may have influenced the representation of individual keyword nodes and cluster boundaries.

Fifth, bibliometric analysis is a quantitative mapping approach that cannot substitute for systematic reviews or experimental validation, nor can it capture knowledge exchange occurring through preprints and informal channels.

Sixth, Author keywords were retained in the forms indexed by the WoS CC. Consequently, lexical variants, including singular and plural forms and alternative spellings such as “waste water” and “wastewater”, may have been represented as separate nodes, potentially fragmenting term frequencies and local co-occurrence patterns. LLR labels were therefore interpreted conservatively, and semantically overlapping ARG- and wastewater-related terms were synthesized at the broader thematic level rather than treated as independent research directions. Future updates should apply an a priori keyword thesaurus and report a sensitivity analysis based on harmonized terms.

Seventh, the division of the publication trajectory into three periods was descriptive and was not validated using segmented regression or structural break testing. The relatively short series of complete annual observations, particularly the three-year intermediate period, would make a two-breakpoint model sensitive to individual-year variation. Future studies based on a longer and fully completed time series should test temporal changes using formal change-point methods appropriate for publication-count data.

Eighth, author and institution disambiguation may be incomplete. Variations in initials, name order, institutional abbreviations, mergers, and affiliation changes may split the same entity across multiple nodes or combine distinct entities with similar names. Although the records and leading nodes were manually checked, residual disambiguation errors cannot be excluded.

Lastly, the bibliometric analysis was not validated against external clinical surveillance databases, wastewater-monitoring programs, policy implementation records, or public-health intervention datasets. Consequently, the emergence and co-occurrence of public-health and One Health terminology should not be interpreted as evidence that operational integration has already occurred or that wastewater metagenomic surveillance has produced measurable health outcomes.

## 5. Conclusions

This study performed a systematic bibliometric analysis of 1161 publications from the Web of Science Core Collection, using CiteSpace to map research on metagenomic surveillance of AMR and pathogenic microorganisms in wastewater systems from 2010 to 2026.

The field has undergone substantial growth, transitioning from an initial exploratory phase centered on ARG detection in activated sludge to a phase of rapid expansion in which over 70% of total output is concentrated in the most recent five years. This growth trajectory reflects the combined effects of declining sequencing costs, maturation of bioinformatics infrastructure, increased international policy attention to environmental AMR, and the expansion of wastewater-based epidemiological surveillance following the COVID-19 pandemic.

The thematic landscape has progressively broadened from foundational studies on specific resistance types and treatment processes to encompass resistome profiling, virulence factor analysis, pathogen surveillance, and integration of wastewater monitoring with the One Health framework. Keyword timeline and cluster analyses indicate that this transformation is underway but remains in its early stages, with the environmental science dimension considerably more mature than its public-health counterpart. The intellectual base of the field rests on three pillars: global wastewater surveillance empirical studies, bioinformatics tools and resistance gene databases, and foundational work on ARG ecology in wastewater systems. Current research attention is focused on analytical quality, computational tool development, and broader water environment surveillance.

Collaboration network analyses revealed concentrated output dominated by China and the United States with limited international bridging, fragmented partnerships at the institutional and author levels, and a pronounced underrepresentation of the Global South. Addressing these structural imbalances, together with the standardization of metagenomic surveillance protocols, the integration of metagenomic data with epidemiological and clinical datasets, the development of quantitative risk assessment frameworks, and the establishment of transnational data-sharing platforms, will be essential for transforming wastewater metagenomic surveillance from an environmental characterization tool into an operational component of public-health early-warning systems. Collectively, the bibliometric map presented here provides a strategic reference for prioritizing the methodological, translational, and structural investments needed to turn wastewater metagenomic surveillance into an actionable instrument against antimicrobial resistance.

## Data Availability

The original contributions presented in this study are included in the article/Supplementary Material. Further inquiries can be directed to the corresponding author.

## Supplementary Materials

The following supporting information can be downloaded at: https://www.mdpi.com/article/10.3390/microorganisms14071583/s1.

## References

[1] Murray C.J.L., Ikuta K.S., Sharara F., Swetschinski L., Aguilar G.R., Gray A., Han C., Bisignano C., Rao P., Wool E. (2022). Global burden of bacterial antimicrobial resistance in 2019: A systematic analysis. Lancet.

[2] Larsson D.G.J., Flach C.-F. (2022). Antibiotic resistance in the environment. Nat. Rev. Microbiol..

[3] Bradshaw A. (2025). Mobile genetic elements and wastewater treatment: Contaminants of emerging concern, climate change, and trophic transmission. Front. Microbiol..

[4] Pärnänen K.M.M., Narciso-da-Rocha C., Kneis D., Berendonk T.U., Cacace D., Do T.T., Elpers C., Fatta-Kassinos D., Henriques I., Jaeger T. (2019). Antibiotic resistance in European wastewater treatment plants mirrors the pattern of clinical antibiotic resistance prevalence. Sci. Adv..

[5] Wang J., Chu L., Wojnárovits L., Takács E. (2020). Occurrence and fate of antibiotics, antibiotic resistant genes (ARGs) and antibiotic resistant bacteria (ARB) in municipal wastewater treatment plant: An overview. Sci. Total Environ..

[6] Zhu C., Wu L., Ning D., Tian R., Gao S., Zhang B., Zhao J., Zhang Y., Xiao N., Wang Y. (2025). Global diversity and distribution of antibiotic resistance genes in human wastewater treatment systems. Nat. Commun..

[7] Guo J., Li J., Chen H., Bond P.L., Yuan Z. (2017). Metagenomic analysis reveals wastewater treatment plants as hotspots of antibiotic resistance genes and mobile genetic elements. Water Res..

[8] Berglund F., Ebmeyer S., Kristiansson E., Larsson D.G.J. (2023). Evidence for wastewaters as environments where mobile antibiotic resistance genes emerge. Commun. Biol..

[9] Diamond M.B., Keshaviah A., Bento A.I., Conroy-Ben O., Driver E.M., Ensor K.B., Halden R.U., Hopkins L.P., Kuhn K.G., Moe C.L. (2022). Wastewater surveillance of pathogens can inform public health responses. Nat. Med..

[10] Munk P., Brinch C., Møller F.D., Petersen T.N., Hendriksen R.S., Seyfarth A.M., Kjeldgaard J.S., Svendsen C.A., van Bunnik B., Berglund F. (2022). Genomic analysis of sewage from 101 countries reveals global landscape of antimicrobial resistance. Nat. Commun..

[11] Paul D., Talukdar D., Kapuganti R.S., Gupta V., Narendrakumar L., Jana P., Kumar P., Singh J., Kumari S., Basak C. (2025). Antibiotic contamination and antimicrobial resistance dynamics in the urban sewage microbiome in India. Nat. Commun..

[12] Karaolia P., Vasileiadis S., Michael S.G., Karpouzas D.G., Fatta-Kassinos D. (2021). Shotgun metagenomics assessment of the resistome, mobilome, pathogen dynamics and their ecological control modes in full-scale urban wastewater treatment plants. J. Hazard. Mater..

[13] Angers-Loustau A., Petrillo M., Bengtsson-Palme J., Berendonk T., Blais B., Chan K.-G., Coque T.M., Hammer P., Heß S., Kagkli D.M. (2018). The challenges of designing a benchmark strategy for bioinformatics pipelines in the identification of antimicrobial resistance determinants using next generation sequencing technologies. F1000Research.

[14] Hendriksen R.S., Munk P., Njage P., van Bunnik B., McNally L., Lukjancenko O., Röder T., Nieuwenhuijse D., Pedersen S.K., Kjeldgaard J. (2019). Global monitoring of antimicrobial resistance based on metagenomics analyses of urban sewage. Nat. Commun..

[15] Zhang A.-N., Gaston J.M., Dai C.L., Zhao S., Poyet M., Groussin M., Yin X., Li L.-G., van Loosdrecht M.C.M., Topp E. (2021). An omics-based framework for assessing the health risk of antimicrobial resistance genes. Nat. Commun..

[16] Roy G., Prifti E., Belda E., Zucker J.-D. (2024). Deep learning methods in metagenomics: A review. Microb. Genom..

[17] Parkins M.D., Lee B.E., Acosta N., Bautista M., Hubert C.R.J., Hrudey S.E., Frankowski K., Pang X.-L. (2024). Wastewater-based surveillance as a tool for public health action: SARS-CoV-2 and beyond. Clin. Microbiol. Rev..

[18] Wang R., Ji M., Zhai H., Guo Y., Liu Y. (2021). Occurrence of antibiotics and antibiotic resistance genes in WWTP effluent-receiving water bodies and reclaimed wastewater treatment plants. Sci. Total Environ..

[19] Mendelson M., Lewnard J.A., Sharland M., Cook A., Pouwels K.B., Alimi Y., Mpundu M., Wesangula E., Weese J.S., Røttingen J.-A. (2024). Ensuring progress on sustainable access to effective antibiotics at the 2024 UN General Assembly: A target-based approach. Lancet.

[20] Nizeyimana J.C., Wang Y., Gad M., Manzi H.P., Liao M., Hu A. (2026). Impact of Wastewater Treatment Plant Effluents on Antibiotic Resistance Gene Profiles in Receiving Waters: A Bibliometric Analysis. Rev. Environ. Contam. Toxicol..

[21] Hui K., Hu W., Xi B., Yuan Y., Tan W. (2026). A comprehensive bibliometric analysis of research hotspots and thematic trends in emerging contaminants in groundwater (1999–2024). J. Hydrol..

[22] Xing Z., Fu W., Li L., Wu S. (2025). Bibliometric analysis of microplastics research: Advances and future directions (2020–2024). Cont. Shelf Res..

[23] Li Z., Yuan D. (2024). Global performance and trends of research on emerging contaminants in sewage sludge: A Bibliometric Analysis from 1990 to 2023. Ecotoxicol. Environ. Saf..

[24] Garrido-Cardenas J.A., Polo-López M.I., Oller-Alberola I. (2017). Advanced microbial analysis for wastewater quality monitoring: Metagenomics trend. Appl. Microbiol. Biotechnol..

[25] Chen C. (2006). CiteSpace II: Detecting and visualizing emerging trends and transient patterns in scientific literature. J. Am. Soc. Inf. Sci. Technol..

[26] Sweileh W.M., Moh’d Mansour A. (2020). Bibliometric analysis of global research output on antimicrobial resistance in the environment (2000–2019). Glob. Health Res. Policy.

[27] Chen C., Ibekwe-SanJuan F., Hou J. (2010). The structure and dynamics of cocitation clusters: A multiple-perspective cocitation analysis. J. Am. Soc. Inf. Sci. Technol..

[28] Wu N., Li M. (2022). A CiteSpace-Based Analysis of the Development Trends Affecting Clinical Research Nurses in China: A Systematic Review. J. Multidiscip. Healthc..

[29] Jeong Y.K., Song M., Ding Y. (2014). Content-based author co-citation analysis. J. Informetr..

[30] Ju F., Beck K., Yin X., Maccagnan A., McArdell C.S., Singer H.P., Johnson D.R., Zhang T., Bürgmann H. (2019). Wastewater treatment plant resistomes are shaped by bacterial composition, genetic exchange, and upregulated expression in the effluent microbiomes. ISME J..

[31] Li B., Yang Y., Ma L., Ju F., Guo F., Tiedje J.M., Zhang T. (2015). Metagenomic and network analysis reveal wide distribution and co-occurrence of environmental antibiotic resistance genes. ISME J..

[32] Rizzo L., Manaia C., Merlin C., Schwartz T., Dagot C., Ploy M.C., Michael I., Fatta-Kassinos D. (2013). Urban wastewater treatment plants as hotspots for antibiotic resistant bacteria and genes spread into the environment: A review. Sci. Total Environ..

[33] Yang Y., Li B., Zou S., Fang H.H.P., Zhang T. (2014). Fate of antibiotic resistance genes in sewage treatment plant revealed by metagenomic approach. Water Res..

[34] Cacace D., Fatta-Kassinos D., Manaia C.M., Cytryn E., Kreuzinger N., Rizzo L., Karaolia P., Schwartz T., Alexander J., Merlin C. (2019). Antibiotic resistance genes in treated wastewater and in the receiving water bodies: A pan-European survey of urban settings. Water Res..

[35] Goodwin S., McPherson J.D., McCombie W.R. (2016). Coming of age: Ten years of next-generation sequencing technologies. Nat. Rev. Genet..

[36] Mardis E.R. (2017). DNA sequencing technologies: 2006–2016. Nat. Protoc..

[37] World Health Organization (2015). Global Action Plan on Antimicrobial Resistance.

[38] Mtetwa H.N., Amoah I.D., Kumari S., Bux F., Reddy P. (2021). Wastewater-Based Surveillance of Antibiotic Resistance Genes Associated with Tuberculosis Treatment Regimen in KwaZulu Natal, South Africa. Antibiotics.

[39] Megantara I., Sylviana N., Amira P.A., Pradini G.W., Krissanti I., Lesmana R. (2023). Potential of waterbodies as a reservoir of *Escherichia coli* pathogens and the spread of antibiotic resistance in the Indonesian aquatic environment. J. Water Sanit. Hyg. Dev..

[40] Maugeri A., Barchitta M., Basile G., Agodi A. (2024). Public and Research Interest in Telemedicine from 2017 to 2022: Infodemiology Study of Google Trends Data and Bibliometric Analysis of Scientific Literature. J. Med. Internet Res..

[41] Taneja N., Sharma M. (2019). Antimicrobial resistance in the environment: The Indian scenario. Indian J. Med. Res..

[42] Dua J., Singh V.K., Lathabai H.H. (2023). Measuring and characterizing international collaboration patterns in Indian scientific research. Scientometrics.

[43] He S., Shrestha P., Henry A.D., Legido-Quigley H. (2023). Leveraging collaborative research networks against antimicrobial resistance in Asia. Front. Public Health.

[44] Vikesland P.J., Pruden A., Alvarez P.J.J., Aga D., Bürgmann H., Li X.-d., Manaia C.M., Nambi I., Wigginton K., Zhang T. (2017). Toward a Comprehensive Strategy to Mitigate Dissemination of Environmental Sources of Antibiotic Resistance. Environ. Sci. Technol..

[45] Alcock B.P., Raphenya A.R., Lau T.T.Y., Tsang K.K., Bouchard M., Edalatmand A., Huynh W., Nguyen A.-L.V., Cheng A.A., Liu S. (2020). CARD 2020: Antibiotic resistome surveillance with the comprehensive antibiotic resistance database. Nucleic Acids Res..

[46] Arango-Argoty G., Garner E., Pruden A., Heath L.S., Vikesland P., Zhang L. (2018). DeepARG: A deep learning approach for predicting antibiotic resistance genes from metagenomic data. Microbiome.

[47] Martínez J.L., Coque T.M., Baquero F. (2015). What is a resistance gene? Ranking risk in resistomes. Nat. Rev. Microbiol..

[48] Su J.-Q., An X.-L., Li B., Chen Q.-L., Gillings M.R., Chen H., Zhang T., Zhu Y.-G. (2017). Metagenomics of urban sewage identifies an extensively shared antibiotic resistome in China. Microbiome.

[49] Zhang Z., Zhang Q., Wang T., Xu N., Lu T., Hong W., Penuelas J., Gillings M., Wang M., Gao W. (2022). Assessment of global health risk of antibiotic resistance genes. Nat. Commun..

[50] Wood D.E., Lu J., Langmead B. (2019). Improved metagenomic analysis with Kraken 2. Genome Biol..

[51] Yin X., Jiang X.-T., Chai B., Li L., Yang Y., Cole J.R., Tiedje J.M., Zhang T. (2018). ARGs-OAP v2.0 with an expanded SARG database and Hidden Markov Models for enhancement characterization and quantification of antibiotic resistance genes in environmental metagenomes. Bioinformatics.

[52] Liepa E., Ustinova M., Gudra D., Roga A., Kalnina I., Dejus B., Dejus S., Strods M., Tomsone L.E., Kibilds J. (2026). Urban Wastewater Metagenomics Reveals the Antibiotic Resistance Gene Distribution Across Latvian Municipalities. Microorganisms.

[53] Ma Y., Wu N., Zhang T., Li Y., Cao L., Zhang P., Zhang Z., Zhu T., Zhang C. (2024). The microbiome, resistome, and their co-evolution in sewage at a hospital for infectious diseases in Shanghai, China. Microbiol. Spectr..

[54] Zhu L., Yuan L., Shuai X.-Y., Lin Z.-J., Sun Y.-J., Zhou Z.-C., Meng L.-X., Ju F., Chen H. (2023). Deciphering basic and key traits of antibiotic resistome in influent and effluent of hospital wastewater treatment systems. Water Res..

[55] Baker-Austin C., Wright M.S., Stepanauskas R., McArthur J.V. (2006). Co-selection of antibiotic and metal resistance. Trends Microbiol..

[56] Pal C., Bengtsson-Palme J., Kristiansson E., Larsson D.G.J. (2015). Co-occurrence of resistance genes to antibiotics, biocides and metals reveals novel insights into their co-selection potential. BMC Genom..

[57] Di Cesare A., Eckert E.M., D’Urso S., Bertoni R., Gillan D.C., Wattiez R., Corno G. (2016). Co-occurrence of integrase 1, antibiotic and heavy metal resistance genes in municipal wastewater treatment plants. Water Res..

[58] Gullberg E., Cao S., Berg O.G., Ilbäck C., Sandegren L., Hughes D., Andersson D.I. (2011). Selection of Resistant Bacteria at Very Low Antibiotic Concentrations. PLoS Pathog..

[59] Pal C., Bengtsson-Palme J., Rensing C., Kristiansson E., Larsson D.G.J. (2014). BacMet: Antibacterial biocide and metal resistance genes database. Nucleic Acids Res..

[60] Calero-Cáceres W., Ye M., Balcázar J.L. (2019). Bacteriophages as Environmental Reservoirs of Antibiotic Resistance. Trends Microbiol..

[61] Wang M., Xiong W., Liu P., Xie X., Zeng J., Sun Y., Zeng Z. (2018). Metagenomic Insights Into the Contribution of Phages to Antibiotic Resistance in Water Samples Related to Swine Feedlot Wastewater Treatment. Front. Microbiol..

[62] Worp N., Nieuwenhuijse D.F., Izquierdo-Lara R.W., Schapendonk C.M.E., Brinch C., Jensen E.E.B., Munk P., Hendriksen R.S., Aarestrup F., Munnink B.B.O. (2025). Unveiling the global urban virome through wastewater metagenomics. Nat. Commun..

[63] Blaak H., Witteveen S., de Haan A., van Santen-Verheuvel M.G., Kemper M.A., de Roda Husman A.M., Hendrickx A.P.A., Schmitt H. (2026). The Dutch CPE Surveillance Study Group. Comparative Genomics of Human- and Wastewater-Derived CPE Isolates in The Netherlands Reveals Shared and Complementary Characteristics. Microorganisms.

[64] Bivins A., North D., Ahmad A., Ahmed W., Alm E., Been F., Bhattacharya P., Bijlsma L., Boehm A.B., Brown J. (2020). Wastewater-Based Epidemiology: Global Collaborative to Maximize Contributions in the Fight Against COVID-19. Environ. Sci. Technol..

[65] Bowes D.A., Driver E.M., Choi P.M., Barcelo D., Beamer P.I. (2024). Wastewater-based epidemiology to assess environmentally influenced disease. J. Expo. Sci. Environ. Epidemiol..

[66] Choi P.M., Tscharke B.J., Donner E., O’Brien J.W., Grant S.C., Kaserzon S.L., Mackie R., O’Malley E., Crosbie N.D., Thomas K.V. (2018). Wastewater-based epidemiology biomarkers: Past, present and future. TrAC Trends Anal. Chem..

[67] United Nations Environment Programme (2023). Bracing for Superbugs.

[68] Cherian J.J., Rahi M., Singh S., Reddy S.E., Gupta Y.K., Katoch V.M., Kumar V., Selvaraj S., Das P., Gangakhedkar R.R. (2021). India’s Road to Independence in Manufacturing Active Pharmaceutical Ingredients: Focus on Essential Medicines. Economies.

[69] Panteli D., Anderson M., Fieldman T., Baraldi E., Tängdén T., Vogler S., Årdal C., Mossialos E. (2024). Policy options for sustainable access to off-patent antibiotics in Europe. npj Antimicrob. Resist..

[70] Aarestrup F.M., Woolhouse M.E.J. (2020). Using sewage for surveillance of antimicrobial resistance. Science.

[71] Sims N., Kasprzyk-Hordern B. (2020). Future perspectives of wastewater-based epidemiology: Monitoring infectious disease spread and resistance to the community level. Environ. Int..

[72] Kleinberg J. (2003). Bursty and Hierarchical Structure in Streams. Data Min. Knowl. Discov..

[73] Nguyen A.Q., Vu H.P., Nguyen L.N., Wang Q., Djordjevic S.P., Donner E., Yin H., Nghiem L.D. (2021). Monitoring antibiotic resistance genes in wastewater treatment: Current strategies and future challenges. Sci. Total Environ..

[74] Partridge S.R., Kwong S.M., Firth N., Jensen S.O. (2018). Mobile Genetic Elements Associated with Antimicrobial Resistance. Clin. Microbiol. Rev..

[75] Hardwick S.A., Chen W.Y., Wong T., Kanakamedala B.S., Deveson I.W., Ongley S.E., Santini N.S., Marcellin E., Smith M.A., Nielsen L.K. (2018). Synthetic microbe communities provide internal reference standards for metagenome sequencing and analysis. Nat. Commun..

[76] Ray K. (2017). Human faecal sample processing in metagenomic studies: Striving for standards. Nat. Rev. Gastroenterol. Hepatol..

[77] Schlaberg R., Chiu C.Y., Miller S., Procop G.W., Weinstock G. (2017). Validation of Metagenomic Next-Generation Sequencing Tests for Universal Pathogen Detection. Arch. Pathol. Lab. Med..

[78] Che Y., Xia Y., Liu L., Li A.-D., Yang Y., Zhang T. (2019). Mobile antibiotic resistome in wastewater treatment plants revealed by Nanopore metagenomic sequencing. Microbiome.

[79] Quick J., Loman N.J., Duraffour S., Simpson J.T., Severi E., Cowley L., Bore J.A., Koundouno R., Dudas G., Mikhail A. (2016). Real-time, portable genome sequencing for Ebola surveillance. Nature.

[80] Liguori K., Keenum I., Davis B.C., Calarco J., Milligan E., Harwood V.J., Pruden A. (2022). Antimicrobial Resistance Monitoring of Water Environments: A Framework for Standardized Methods and Quality Control. Environ. Sci. Technol..

[81] Becsei Á., Fuschi A., Otani S., Kant R., Weinstein I., Alba P., Stéger J., Visontai D., Brinch C., de Graaf M. (2024). Time-series sewage metagenomics distinguishes seasonal, human-derived and environmental microbial communities potentially allowing source-attributed surveillance. Nat. Commun..

[82] Majeed H.J., Riquelme M.V., Davis B.C., Gupta S., Angeles L., Aga D.S., Garner E., Pruden A., Vikesland P.J. (2021). Evaluation of Metagenomic-Enabled Antibiotic Resistance Surveillance at a Conventional Wastewater Treatment Plant. Front. Microbiol..

